# Magnetic Abrasive Machining of Difficult-to-Cut Materials for Ultra-High-Speed Machining of AISI 304 Bars

**DOI:** 10.3390/ma10091029

**Published:** 2017-09-04

**Authors:** Rui Wang, Pyo Lim, Lida Heng, Sang Don Mun

**Affiliations:** Division of Mechanical Design Engineering, Chonbuk National University, 664-14, Duckjin-gu, Jeonju 561-756, Korea; wangruiaa@hotmail.com (R.W.); satanr@hanmail.net (P.L.); henglida1@gmail.com (L.H.)

**Keywords:** ultra-high-speed magnetic abrasive machining (UHSMAM), micro-diameter machining, surface roughness, AISI 304 bar, regression analysis, atomic force microscope (AFM)

## Abstract

This research proposes an optimized magnetic abrasive machining process that uses an ultra-high-speed system to perform precision machining on a workpiece. The system can process several microns of material, either for machining surface roughness or for machining a workpiece for a precise micro-diameter. The stainless steel workpieces have been machined using an ultra-high-speed magnetic abrasive machining (UHSMAM) process. The experiments were performed analyzing the accuracy of the machined workpiece diameter, using response surface methodology. The results obtained after machining have been analyzed to determine the effect of different process parameters such as machining speed, machining time, machining frequencies, inert gas in/out, magnetic pole types, and magnetic abrasive mesh size for the individual workpiece, as well as to study various interaction effects that may significantly affect the machining performance of the process. The obtained outcomes of the analysis for different workpieces have been critically compared to understand the effect of the considered process parameters based on the resulting mechanical properties. Regression analysis was used to confirm the stability of the micro-diameter and the processing efficiency. Atomic force microscope (AFM) micrographs were also obtained to study the surface morphology of the precision-machined workpiece.

## 1. Introduction

Amidst the remarkable advancements in industrial technologies, intensive attempts have been made to achieve superior mechanical properties and performances. Currently, nonconventional materials such as stainless steels, titanium alloys, tungsten, and various composites are widely used in industries because these materials have special characteristics such as high hardness, heat resistance, wear resistance, and high strength. However, most of these materials have poor machining accuracy and machinability. These materials are difficult to process and are called difficult-to-cut materials [1,2].

Difficult-to-cut materials, stainless steel in particular, are frequently demanded for applications with harsh operational conditions or in applications in the fields of semiconductors, biotechnology, medical, and aeronautical/aerospace industries, where ultra-precision parts are necessary. To keep pace with these trends, the machining/polishing field continuously adopts more advanced methods for processing materials [3,4]. Among these special-purpose polishing methods is magnetic abrasive finishing [5,6,7], which has been widely put into practice as an ultra-precision technology. Magnetic abrasive finishing uses a combination of magnetic and abrasive materials in a magnetic field to generate a fine abrading force on the surface of parts or objects [8,9]. In this study, we demonstrate methods to obtain a substantial increase in magnetic abrasive processing speed using an ultra-high-speed system optimized to outperform conventional magnetic abrasive polishing [10,11]. The magnetic abrasive machining (MAM) is a machining technique in which a magnetic field is used to force on abrasive particles against the target accuracy, and the cutting tool is a group of magnetic abrasive particles, the cutting force is controlled by the magnetic field in the working gap [12]; in particular, progress is demonstrated in the precision machining of micro-diameters and in minimizing surface roughness with a significantly reduced processing time and maximized processing efficiency. It is proposed that the results of this study enable true micron-scale machining of metals that cannot be obtained with traditional processing methods.

In the present study, we applied ultra-high-speed magnetic abrasive machining (UHSMAM) processes in the precision machining of stainless steel (AISI 304). The experiments were performed based on the response surface methodology to enhance the machining performance and productivity. The aim is to analyze the effect of important process parameters such as machining speed, machining time, machining frequencies, inert gas in/out, magnetic pole types, and magnetic abrasive mesh size. In order to forecast the machining surface roughness and micro-diameter of workpiece before the machining process, an experiment model was applied using the Minitab software. The generation of the model consists of two stages, training and testing based on experimental data. Thus, in this study, a model with six inputs and two outputs has been considered. An appropriate setup was designed and fabricated. The Taguchi method is the most significant and useful parameter in taking the target and variation into account when comparing two sets of samples, as opposed to comparing the mean alone. Thus, the Taguchi method using S/N ratio is applied to perform uniform machining. On the two response factors, the surface roughness and micro-diameter, the parameters are optimized by analysis of variance (ANOVA) of the S/N ratio. The atomic force microscope (AFM) micrographs were used to further analyze the results.

## 2. Principle of the UHSMAM Process

### 2.1. Process Principle

**Figure 1:**
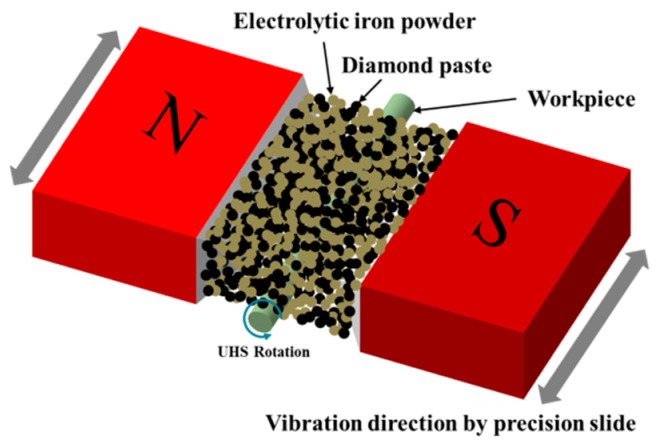
Schematic of the processing principles of cylindrical magnetic abrasive machining.

A method of external surface machining of bars using ultra-high-speed magnetic abrasive machining (UHSMAM) has been proposed for AISI 304 materials. The machining speed is the most critical parameter in the machining process [13]. In ultra-high-speed machining, centrifugal force tends to cause the displacement of the lubricant from the finishing areas, which in turn causes the mixed-type magnetic abrasive to adhere to the workpiece surface due to friction [10]. Figure 1 shows a schematic of the magnetic abrasive machining process, where an unbounded type magnetic abrasive (electrolytic iron powder + diamond paste) is filled between the N and S magnetic poles (180° aligned arrangement) to form a magnetic abrasive brush that parallels the direction of the magnetic field lines. The targeted round workpiece is inserted into the magnetic abrasive brush and then rotated at ultra-high-speeds, while the magnets are vibrated in the axial direction of the workpiece. The machining force affecting the target object is exerted by magnetic particles on its surface perpendicular to the direction of magnetic force lines, accordingly causing the desired abrasion, thereby simultaneously completing the processing of the surface roughness and the micro-diameter of the same portion of the workpiece.

**Figure 2:**
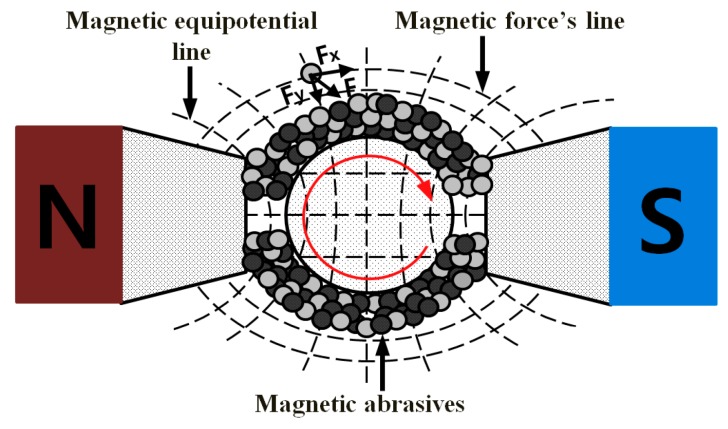
Two-dimensional magnetic force field lines holding particles operating on the workpiece [11].

Figure 2 shows the distribution of magnetic particles in the alternating magnetic field. A magnetic particle along the direction of the magnetic equipotential line generates a force $F_{x}$, and a particle perpendicular to the magnetic force line direction generates a force $F_{y}$. The magnetic force F that acts on a magnetic particle is calculated using the following formula [14]:

(1) $$F_{X}=X_{FP}\mu_{0}VH\left(\frac{dH}{dX}\right)\;\&\;F_{Y}=X_{FP}\mu_{0}VH\left(\frac{dH}{dY}\right).$$

Here, V is the volume of magnetic particles, $X_{FP}$ is the susceptibility of ferromagnetic particles, $\mu_{0}$ ($m^{3}/\mathrm{kg}$) is the permeability of free space, and H (T) and $\frac{dH}{dX}$ and $\frac{dH}{dY}$ (A/$m^{f}$) are the magnetic field intensity and the gradients magnetic field strength in the *X* and *Y* directions [15].

On the external surface of the workpiece, the resultant magnetic force F is generated by the magnetic abrasive and the magnetic particles attempt to follow the force lines of the magnetic force. If the tangential component of the magnetic force acting on the magnetic abrasive is larger than the frictional force between the magnetic abrasive and the external surface of the workpiece, then the magnetic abrasive exhibits a smooth relative motion against the external surface when the workpiece is rotated at ultra-high-speeds. Manipulating the poles along the workpiece axis causes the magnetic abrasive to move in the axial direction following the pole motion, effectively machining the external surface and machining the micro-diameter in the particular case of bars.

### 2.2. Magnetic Abrasives

Magnetic abrasives act as a cutting tool in the magnetic abrasive machining process. They consist of ferrous particles and non-ferrous abrasives. In this study, the magnetic abrasives are formed by mixing diamond paste (mesh size 0.5 μm, and 1 μm) and electrolytic iron powder (mesh size 200 μm). The mixed type abrasives adopted for the experiment were obtained by mechanically mixing diamond paste and electrolytic iron particles with 0.1 mL of grinding fluid as a lubricant. The mixed abrasives were formed under the action of the magnetic field on the powerful magnetic brush, adhering to the gap between the magnetic poles and the workpiece surface.

## 3. Experimental Details

**Figure 3:**
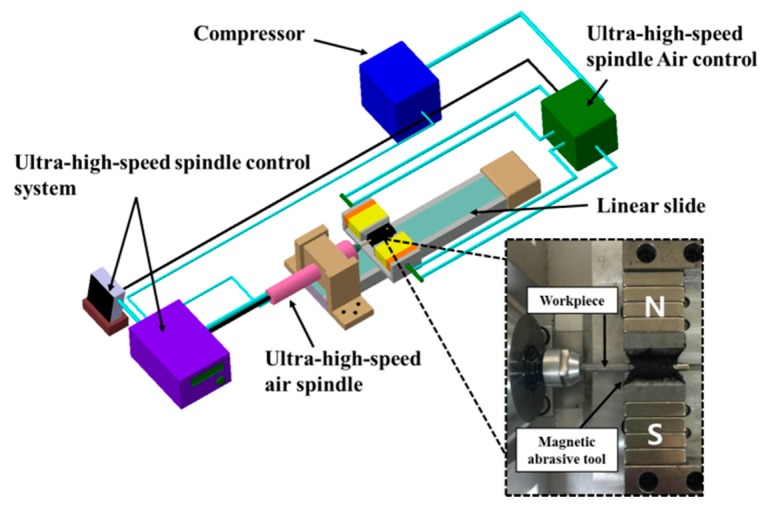
Photographic view of the ultra-high-speed magnetic abrasive machining (UHSMAM) experimental setup.

As shown in Figure 3, the apparatus for magnetic abrasive machining is designed in such a way that a fine AISI 304 round bar is positioned between the magnetic poles. Table 1 shows the mechanical properties and chemical composition of AISI 304. The bar is then rotated with an ultra-high-speed of up to 80,000 rpm using an ultra-high-speed air spindle control system, consisting of an ultra-high-speed air spindle (NSK Air bearing spindle, NRA-5080), the rotor of the air turbine was used because it was specifically designed to maximize ultra-high-speed on the ultra-high-speed spindle during the machining process. The rotor is rotated by the velocity of the air stream, making this ultra-high-speed spindle perfect for applications requiring operation using the principle of compressed air blown. This ultra-high-speed spindle has almost no vibration and extremely high accuracy for extremely high precision machining, even at ultra-high-speed rotation. Simultaneously, a 5 Hz or 10 Hz vibration is applied to allow axial control of the bar machining. For this study, a pneumatic spindle was used to ensure the AISI 304 bar could undergo a very high number of revolutions. The Nd–Fe–B permanent magnet (size: 20 mm × 10 mm × 12 mm, magnetic flux density: 0.52 T) was used to produce the magnetic field. The desired magnetic field in the machining area is generated by permanent magnets attached to a steel yoke.

### Selection of Process Parameters

**Figure 4:**
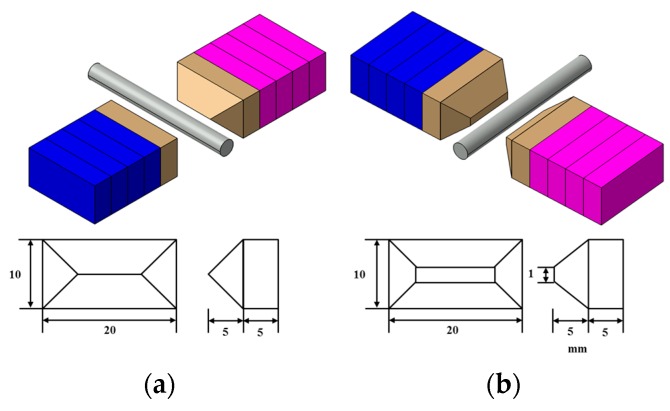
Experimental conditions for the two kinds of magnetic pole types. (**a**) The sharp shape; (**b**) a 1 mm linear shape.

In the present study, an experimental investigation was carried out to study the effects of important process parameters in the UHSMAM process for machining AISI 304 materials using the Taguchi experimental design. Based on preliminary experiments and the available literature on the MAM process, the key process parameters and their levels that strongly influence the process outcomes were identified. In this magnetic abrasive apparatus, the desired result is achieved by placing a cylindrical object in the magnetic pole area and rotating it at a rotational speed of 1000–80,000 rpm under a 0–10 Hz range of vibrational conditions. For the magnetic poles, as shown in Figure 4, SS 400 steel materials was chosen due to its relative ease of fabrication and because it is considered a very strong magnetic material in virtue of its specially designed shape and the distribution of magnetic force. Two types of magnetic pole configurations were studied in order to compare their processing characteristics: (a) a sharp shape and (b) a 1 mm linear shape. Mixed-type magnetic abrasives with diamond particles of different diameters were inserted between the poles. A cylindrical object to be processed is inserted into the grainy brush, and rotational and vibrational movements were induced simultaneously to confirm the system’s processing characteristics. An important consideration for ultra-high-speed processing conditions is that, if the workpiece temperature is too high, it will produce an acidification layer and thus affect the processing. During the machining process, argon gas was injected into the machined part to improve the machining efficiency and to parameterize the influence of the argon gas input conditions.

In order to analyze the performance of the machining process on the surface of the AISI 304 bar, six process parameters were selected, including four different workpiece rotational speeds, four different machining times, two different frequencies, whether or not inert argon gas is injected, two different magnetic pole types, and two different magnetic abrasive particle sizes. Some preliminary experiments were performed to determine the influence of these parameters.

## 4. Results and Discussion

This section presents the experimental data analysis for the UHSMAM process. Table 2 shows the factor level and Taguchi $L_{16}$ orthogonal array [16,17], selected to investigate the effects of the selected process parameters, and its output response, such as the measured surface roughness (Ra) and machining micro-diameter. In order to identify the main influencing process parameters for the UHSMAM process, a standard statistical analysis such as the signal-to-noise ratio and analysis of variance (ANOVA) was carried out on the machining data. The Minitab statistical software was used for the statistical data analysis, and the results are presented in this section.

### 4.1. Effects of Process Parameters on the Machining Surface Roughness

**Figure 5:**
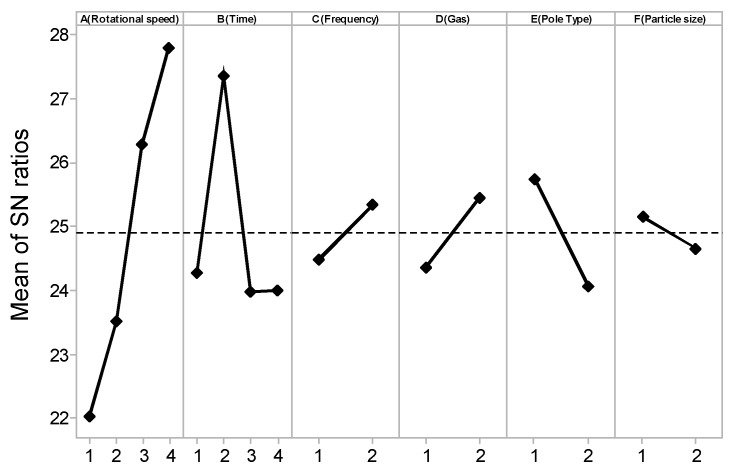
Main effects plot for the S/N ratios of machining surface roughness.

The main effect plot of the process for machining surface roughness is shown in Figure 5. It can be seen from Figure 5 that the machining surface roughness shows a linearly increasing relation with ultra-high rotational speed. This is because at the highest rpm values, the rate at which magnetic abrasive particles hit the workpiece surface increases. Thus, the centrifugal force, which generated by ultra-high-speed magnetic abrasive machining process tends to push the abrasive particles strongly, because the diamond abrasive particle have a very small grain size with high strength cutting edge abrasive, they can produce the smooth surface. Therefore, more peaks are sheared at a higher rpm, resulting in higher surface machining. The processing time is a sensitive condition, as can be seen from Figure 5; 60 s for the processing time shows the highest processing efficiency. Deteriorations of the processing effect with increases in the processing time occur because the increased processing will deepen the processing layer, reducing the processing efficiency. The vibrational speed of 10 Hz has a better processing efficiency than 5 Hz. In the case of higher vibration speed, the magnetic abrasives will largely pass through the workpiece zone at the same time; thus, the surface of the workpiece can effectively improve. Inert gas injection favorably improves the processing effects, because the high temperature generated under ultra-high-speed processing will corrode the surface of the workpiece, thus affecting the surface roughness of the material accuracy. The sharp type can gather more magnetic force and increase the processing efficiency. Particle size has a relatively small effect on surface roughness values, and particles with a diameter of 0.5 μm are better at processing the rod and lending the capacity for fine machining processing. This result can be explained based on the relationship between the high centrifugal force and the small grain size of abrasive particles. When small grain size abrasive particles are used, the surface of workpiece can effectively improve in terms of high centrifugal force.

As shown in Table 3, the regression analysis showed that the machining speed, machining time, and magnetic pole types have a *p*-value close to 0.05. Thus, it can be determined that the experimental data is very significant. However, the frequencies (Hz), inert argon gas, and diamond particle size have *p*-values larger than 0.05. Although these are not the main factors influencing this experiment, we cannot ignore the impact of these factors.

### 4.2. Effects of the Process Parameters on the Machining Micro-Diameter

**Figure 6:**
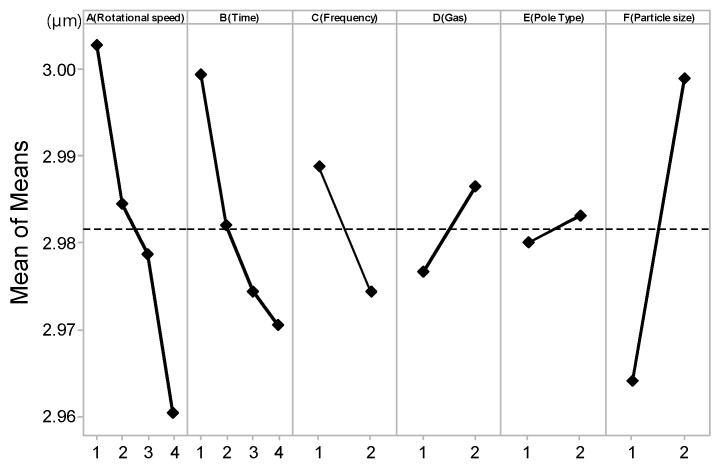
Main effects plot for means of machining micro-diameter.

The main effect plot of the process for machining the diameter is shown in Figure 6. It can be seen from Figure 6 that the machining micro-diameter linearly increases with increases in the ultra-high rotational speed. The result can be explained based on the high centrifugal force, which is generated by the ultra-high-speed magnetic abrasive machining process. During the machining process, high centrifugal forces tend to cause the workpiece to push strongly on the abrasive particle. With an increase in processing time, the diameter removal is increased. The processing efficiency effect in relation to the vibration speeds of 5 Hz and 10 Hz is more prominent. No inert gas injection during the processing effect is favorable, because at ultra-high-speed machining, it is necessary to produce an etching layer due to the occurrence of temporary corrosion. Due to the high heat generated during processing, this corrosive layer softens the surface of the material, thus being more easily removed, thereby maximizing the processing efficiency of the micro-diameter. The processing efficiency with a sharp magnet edge is more favorable than the 1 mm linear type of magnet edge. This is because the sharp type can concentrate field lines, resulting in a greater magnetic force. And the same as the machining surface roughness, this result can be explained based on the relationship between the high centrifugal force and the small grain size of abrasive particles. When small grain size abrasive particles are used, the surface of workpiece can effectively improve in terms of high centrifugal force.

As shown in Table 4, the regression analysis shows that the rotational speed, diamond particle size have *p*-values close to 0.05, meaning that the experimental data is very significant. However, the machining time, frequencies (Hz), inert argon gas, magnetic pole types have *p*-values larger than 0.05. Even though these later parameters are not the most important factors in this experiment, we cannot ignore the impact of these factors.

### 4.3. Optimization of the Objective Function

**Figure 7:**
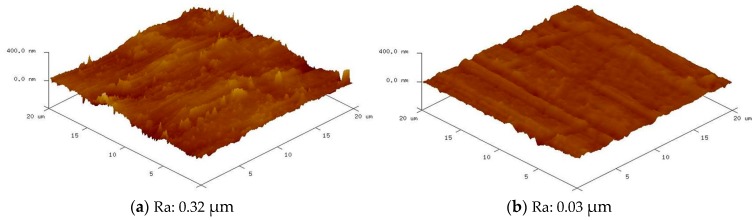
AFM images of the surface conditions (**a**) prior to and (**b**) after machining.

We observe the main plot effects of various process parameters on the machining surface roughness and analyze the machining micro-diameter. Based on this, a set of optimum process parameters were selected and an experiment was performed using this result. The optimum results obtained from the experiment are shown in Table 5 and Table 6. There are some differences in the processing time and diamond particle size, indicating that 60 s is the most appropriate time for machining the surface roughness. When machining micro-diameters, the longer the processing time, the better the processing capacity. Additionally, with regard to the machined surface roughness, the superior processing efficiency of 0.5 μm diamond particles can produce a precision surface. Further, relatively large particles produce a larger processing force and thus are more efficient for reducing the diameters. Figure 7a,b show the AFM images of conditions prior to and after machining the same sample with an optimum set of parameters, respectively. We can see that the scratches and deep grooves were completely removed from the machined surface. The machined surface was significantly smoother than the original sample, and the initial surface toughness of 0.32 μm (Ra) was improved to 0.03 μm (Ra) at 60 s.

By observing the main effect plot, the effect of various process parameters on the machining micro-diameter is analyzed. Based on this analysis, a set of optimum process parameters were selected and an experiment was performed using this data. The optimum result obtained with the experiment is shown in Table 6. In order to ensure the correctness of the regression formula, three workpieces were selected and conducted with the same experiment, each work was tested six times from 0 to 120 s 18 times. The processing data was analyzed again by regression analysis to derive a regression equation. Through this equation, the optimization results can be more effectively utilized, and the processing time can be budgeted according to the desired accuracy.

Machining micro-diameter = −0.5631 + 0.2539 Machining Time − 0.000276 Machining Time ^ 2(2)

S = 466392, R-square = 99.7%.

**Figure 8:**
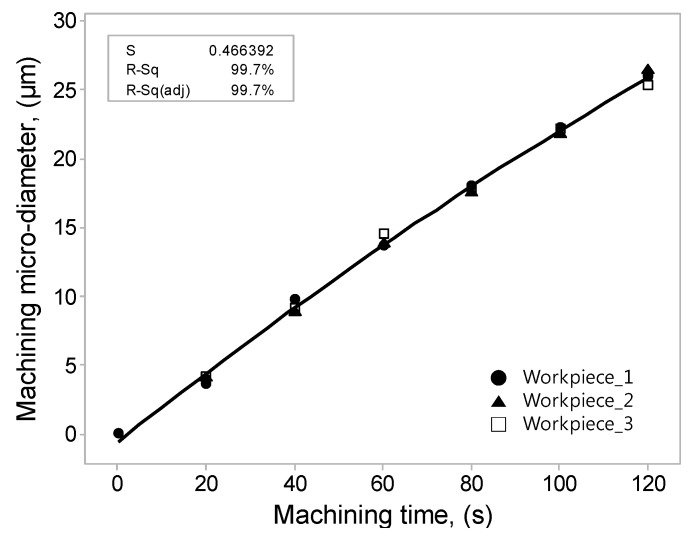
Regression analysis of the machining micro-diameter.

Table 7 shows the analysis of variance for the machining micro-diameter. The analysis of variance performed initially included some insignificant terms which were machined micro-diameter and the analysis of variance was carried out again with significant terms. Figure 8 shows the amount of micro-diameter processing per unit time in a quadratic regression curve, with 20 s fixed intervals, the coefficient of determination is 99.7%, this can more effectively illustrate the feasibility of the regression formula.

## 5. Conclusions

In this study, a difficult-to-cut material consisting of an AISI 304 workpiece was successfully micro-machined using ultra-high-speed magnetic abrasive machining (UHSMAM). Experiments were carried out across a range of input parameters in order to analyze the impacts on surface roughness and machining micro-diameter. The conclusions based on these results are as follows:

1. When machining the surface roughness of the AISI 304 bar, the best machining conditions were found using analysis of variance. Here, the best conditions are a machining speed of 80,000 rpm, 60 s of machining time, a 10 Hz vibrational frequency, inert gas injection, a sharp magnetic pole type, and a 0.5 μm diamond particle mesh size. The best processing conditions yield an exceptionally smooth surface with features smaller than 0.03 μm.
2. For machining, the micro-diameter of the AISI 304 bar, the best conditions are a machining speed of 80,000 rpm, 120 s of machining time, a vibrational frequency of 10 Hz, inert gas injection, a sharp magnetic pole type, and a 0.5 μm diamond particle mesh size. It is known that, as the processing time increases, the processing capacity also increases, and the relatively larger processed particles remove material with greater efficiency. After determining the best processing conditions for machining the micro-diameter of the workpiece, the effective processing efficiency was confirmed via regression analysis. Near the settings of the optimal condition, the amount of processing per unit of time is certain, and the regression equation can be used to budget the production corresponding to the desired processing capacity.

## Figures and Tables

**Table 1:** Mechanical properties and chemical composition of the workpiece.

**Mechanical Properties of AISI 304**								
Workpiece(mm)					Ø 3 × 40 bar			
Provider					JFE Steel Corporation			
Density (g/cm^3^)					7.93			
Electric resistance (Ω × cm)					72 × 10^−6^			
Magnetism					Non-magnetic			
Specific heat (J/Kg × °C)					502			
Young’s modulus (N/mm^2^)					193 × 10^3^			
Ultimate tensile strength (MPa)					515			
Allowable stress (MPa)					At 30 °C, 183			
Initial surface roughness (μm)					0.32			
**Chemical Composition of AISI 304**								
C	Mn	Si	P	S		Cr	Ni	N
0.030	2.0	0.75	0.045	0.030		20.0	12.0	0.10

**Table 2:** Experimental observations.

**Level**	**A**	**B**	**C**	**D**	**E**	**F**
1	5000	30	5	Inject	0	1
2	30,000	60	10	No-inject	1	0.5
3	55,000	90				
4	80,000	120				
**Expt No.**	**Rotational Speed**	**Machining Time**	**Frequency**	**Inert Argon Gas**	**Magnetic Pole Type**	**Diamond Particle Size**
	(rpm)	(s)	(Hz)	(-)	(mm)	(μm)
1	5000	30	5	Inject	0	1
2	5000	60	5	Inject	0	1
3	5000	90	10	Inject	0	0.5
4	5000	120	10	No-inject	1	0.5
5	30,000	30	5	Inject	1	1
6	30,000	60	5	No-inject	1	1
7	30,000	90	10	Inject	0	0.5
8	30,000	120	10	No-inject	0	0.5
9	55,000	30	10	No-inject	0	1
10	55,000	60	10	Inject	0	1
11	55,000	90	5	No-inject	1	0.5
12	55,000	120	5	Inject	1	0.5
13	80,000	30	10	No-inject	1	0.5
14	80,000	60	10	Inject	1	0.5
15	80,000	90	5	No-inject	0	1
16	80,000	120	5	Inject	0	1

**Table 3:** Analysis of variance (ANOVA) for the S/N ratios of the machining surface roughness.

Factors	Degree of Freedom	Sequential Sum of Squares	Adjusted Mean Squares	F-Ratio	*p*-Value
A	3	0.003241	0.001080	21.31	0.003
B	3	0.000819	0.000273	5.38	0.050
C	1	0.000006	0.000006	0.12	0.740
D	1	0.000056	0.000306	1.11	0.340
E	1	0.000306	0.000001	6.04	0.057
F	1	0.000001	0.000051	0.01	0.911
Error	5	0.000253	-	-	-
Total	15	0.004683	-	-	-

**Table 4:** Analysis of variance (ANOVA) of the ratio for the machining micro-diameter.

Factors	Degree of Freedom	Sequential Sum of Squares	Adjusted Mean Squares	F-Ratio	*p*-Value
A	3	0.003671	0.001224	6.85	0.032
B	3	0.001971	0.000657	3.68	0.097
C	1	0.000839	0.000839	4.69	0.082
D	1	0.000388	0.000388	2.17	0.200
E	1	0.000039	0.000039	0.22	0.659
F	1	0.004881	0.004881	27.33	0.003
Error	5	0.000893	0.000179	-	-
Total	15	0.012683	-	-	-

**Table 5:** Optimization results for machining the AISI 304 surface roughness.

A	B	C	D	E	F
(rpm)	(s)	(Hz)	(-)	(mm)	(μm)
80,000	60	10	Inject	0	0.5

**Table 6:** Optimization results for machining the AISI 304 micro-diameter.

A	B	C	D	E	F
(rpm)	(s)	(Hz)	(-)	(mm)	(μm)
80,000	120	10	Inject	0	0.5

**Table 7:** Analysis of variance for the machining micro-diameter.

Factors	Degree of Freedom	Sequential Sum of Squares	Adjusted Mean Squares	F-Ratio	*p*-Value
Regression	1	550.827	550.827	1526.71	0.000
Error	5	1.804	0.361	-	-
Total	6	552.631	-	-	-
